# Association between war-related traumatic events and blood pressure trajectory: a population-based study among the mid-aged and older Palestinian adults living in Gaza

**DOI:** 10.3389/fpubh.2023.1073284

**Published:** 2023-06-15

**Authors:** Majed Jebril, Mohsen Mazidi, Xin Liu, Mi Baibing, Heba Arafat, Zumin Shi, Youfa Wang

**Affiliations:** ^1^Global Health Institute, School of Public Health, Xi'an Jiaotong University Health Science Center, Xi'an, Shaanxi, China; ^2^Department of Twin Research and Genetic Epidemiology, King's College London, London, United Kingdom; ^3^Medical Research Council Population Health Research Unit, University of Oxford, Oxford, United Kingdom; ^4^Clinical Trial Service Unit and Epidemiological Studies Unit, Nuffield Department of Population Health, University of Oxford, Oxford, United Kingdom; ^5^Department of Laboratory Medicine, Faculty of Applied Medical Science, Al Azhar University, Gaza, Palestine; ^6^Department of Human Nutrition, College of Health Sciences, QU. Health, Qatar University, Doha, Qatar

**Keywords:** Palestine, war events, blood pressure, adults, traumatic event

## Abstract

**Background:**

Little is known regarding health status in an environment characterized by instability and ongoing war risks. This study investigated hypertension disease burden and associations of war-related traumatic events with blood pressure (BP) trajectory over time amongst mid-aged and older Palestinian adults in Gaza Strip.

**Methods:**

From nine primary healthcare centers, medical records between 2013 and 2019 were collected for 1,000 mid-aged and older Palestinian adults living in Gaza. Multinomial logistic regression analysis examined associations between war-related traumatic events and BP trajectories derived using latent class trajectory analysis (LCTA).

**Results:**

The prevalence of self-reported injury (of participants or their family members), death of a family member, and violence due to house bombing was 51.4%, 54.1%, and 66.5%, respectively. In total, 22.4% and 21.4% of participants had constant-very-high (CVH) systolic BP (SBP) (>160 mmHg) and diastolic BP (DBP) (>95 mmHg), and normal-stable SBP and DBP was found only 54.9% and 52.6%, respectively. Injury (participants or family members), death of a family member, and violence due to house bombing during wars were associated with CVH SBP with odds ratios [95 CI, OR = 1.79 (1.28–2.48), 1.90 (1.36–2.65), and 1.44 (1.01–2.05)], respectively. The corresponding figures were [95 CI, OR = 1.92 (1.36–2.71), 1.90 (1.35–2.68), and 1.62 (1.13–2.38)] for CVH DBP. Living in debt was positively associated with CVH SBP, [95 CI, OR = 2.49 (1.73–3.60)] and CVH DBP, [95 CI, OR = 2.37 (1.63–3.45)].

**Conclusion:**

The disease burden related to war-related traumatic events is high and positively related to adverse BP trajectory among the mid-aged and older Palestinians living in Gaza. Intervention programs are needed to manage and prevent chronic diseases in this vulnerable population.

## 1. Introduction

Many people are exposed to violence, conflict, war, and war-related events worldwide (1). Gaza, Palestine, is a unique place in the world where people have been exposed to four fierce wars over the past 13 years (2). Those wars left 4,160 martyrs and more than 18,300 injured; most victims were civilians (3, 4). The United Nations estimates that approximately 18,000 homes in Gaza were entirely or partially destroyed because of those wars, and some are still in ruins (5). Besides, the post-war repercussions of low living standards, poverty, and livelihood in debt (6). In 2019, Gaza Strip residents' poverty rate was 86% (33% severe poverty) (7, 8). Perhaps living at this poverty level among Gazans would make them live their daily lives dependent on others' debts. Meanwhile, life for the average Palestinian in Gaza is getting more wretched (9).

Often these populations also suffer from the high prevalence of non-communicable diseases (NCDs) and a lack of related medical services (10). Exposure to war-related traumatic events may increase the risk of developing hypertension (11), which has become one of the significant health concerns in Gaza (12). Those stressful experiences in war life may increase circulating catecholamine and cortisol levels and blood pressure (BP) over time (13–15).

Several studies have revealed that frequent exposure to stressful life events is a risk factor for developing post-traumatic stress disorder (PTSD) and hypertension (16–18). A meta-analysis of six cohort studies suggested that chronic exposure to stress may influence increased BP; individuals with stronger responses to stressor events were 21% more likely to develop BP than those with less intense responses (19). Moreover, a systematic review found some evidence that armed conflict is associated with increased coronary heart disease, and cerebrovascular and endocrine diseases, in addition to increased BP (20). A recent narrative review showed that stressors associated with war and conflict have also epigenetic impacts on health (21).

Besides the actual BP level, the overtime changes in BP (i.e., BP trajectory) could be used to assess the risk of cardiovascular diseases (CVDs) (22). In Palestine, no previous study has examined the effect of traumatic life events on BP trajectories, which must be explored in light of experienced living circumstances for a long time. Therefore, monitoring BP trajectories is indispensable for CVDs prevention (23, 24).

This study aimed to investigate the burden of hypertension (as an indicator of NCDs) and the association between war-related traumatic events (injury, death, serious illness, loss of job, business bankruptcy, exposure to violence due to house bombing, living in debt, marital separation, or divorce; and exposure to major disasters during wars) with BP trajectory over time in the mid-aged and older Palestinian adults living in Gaza Strip. We hypothesized that these war-related life events affect their health and BP trajectory.

## 2. Methods and materials

### 2.1. Study design and participants

This study used a mixed-method study design and was based on a survey conducted in 2019 and historical longitudinal data extracted (for 2013–2019) among residents regularly visiting nine primary healthcare centers (PHCs) of the Ministry of Health (MoH) in Gaza Strip, Palestine. The participants who met the following criteria were enrolled: (1) Palestinian adult males and females (refugees or citizens) who have lived in Gaza Strip for ≥14 years; and (2) Registered at PHCs and have performed multiple anthropometric and biochemical measurement data during 2013 to 2019. Pregnant women and those having missing data were excluded from the study. A total of 1,120 potential residents were invited to participate in our survey. We excluded those without anthropometric data (*n* = 83) and pregnant (*n* = 37). Ultimately, 1,000 participants were included in the analysis (25).

The Xi'an Jiaotong University Health Science Center Ethical Committee approved this research. It was also approved by the Helsinki ethical approval committee affiliated with the Palestinian Health Research Council (PHRC) in Gaza and the Research Department at the Department of Human Resources Development, Ministry of Health, Gaza (PHRC/HC/576/19). All participants provided written informed consent.

### 2.2. Data collection

To enhance the representativeness, we selected the largest nine clinics out of 51 PHCs in Gaza governorates. The participants were selected by stratified random sampling. One to three PHCs were chosen from each governorate, matching each governorate's population size. The data were collected in two phases: (1) The sociodemographic and war-related traumatic events data were collected by face-to-face questionnaire survey administered by trained health workers. (2) The anthropometric measurements, including weight, height, waist circumference, and systolic and diastolic BP, in addition to biochemical parameters, were extracted (from 2013 to 2019) from the electronic health record (E-Health) system by matching the participant's registration ID (25).

Questions on war-related traumatic events were adopted from the Traumatic Life Events Questionnaire (TLEQ) (26). The questions were chosen based on possible war-traumatic life events to be exposed to and are also common among the Palestinian people. All of these events were caused by the three recent wars in Gaza between 2008 and 2020, including nine binary items (yes, or no): (1) Had injury of any family members; (2) Death of any family member; (3) Had serious illness of any family member based on the affliction with any critical diagnosed diseases including cancer, heart attack, stroke, kidney failure, and liver cirrhosis; (4) Had a loss of a job; (5) Had a business bankruptcy; (6) Exposure to violence due to house bombing; (7) Living in debt; (8) Had marital separation or divorce; and (9) Had major disasters that families have been exposed to during wars and caused a real tragedy for them to lose their livelihood by bombing their private properties, i.e., destroying factories, crops, shops, and vehicles.

BP was measured through a calibrated sphygmomanometer after a quiet rest time of 3–5 min, an average measurement of 2–3 BPs, use of an adequately sized cuff placed on a bare arm, and proper patient positioning, including back and heart-level arm support. Each year's average BP measurements were extracted for each participant.

### 2.3. Definitions of hypertension and trajectories of BP

Hypertension was defined as having a documented diagnosis by general practitioners in the PHCs, with measurements of SBP ≥140 mmHg and/or DBP ≥90 mmHg, based on WHO criteria (27).

This study identified BP trajectories using latent class trajectory analysis (LCTA), a method used to identify unobserved trajectory classes in epidemiological data (28). LCTA has the advantage of identifying distinct groups with similar underlying trajectories (29–31). These trajectories can vary in functional form across different-order polynomials, allowing the best-fitting polynomial form to be specified for each trajectory separately, including the coefficients' polynomial order. The Bayesian Information Criterion (BIC) was calculated to evaluate the number of distinct trajectories and choose the best-fit model (32).

### 2.4. Statistical analysis

We conducted descriptive- and in-depth modeling analyses. Continuous variables were summarized as mean values and standard deviations, while categorical variables were described as frequency and percentage. To compare categorical data and continuous variables, respectively, chi-square and *t*-tests were used. One-way variance analysis (ANOVA) was used to compare the demographic characteristics between groups with three or more groups.

The dynamic patterns (trajectories) in SBP and DBP between 2013 and 2019 were derived using LCTA, and their associations with war-related traumatic events were analyzed using multinomial logistic regression analysis. The model was adjusted for potential covariates. Principal components analysis (PCA) was used to develop a composite score based on nine war-related traumatic events. We have obtained the PCA for all nine war-related traumatic events based on the number of observations (*N* = 1,000) by calculating the principal component score based on the loadings of each traumatic event. Then, we calculated the estimated mean for both SBP and DBP by Mean ± SD.

All analyses were conducted using STATA software (version 14.0) and SAS (version 9.4). *P* < 0.05 was considered statistically significant.

## 3. Results

### 3.1. Characteristics of the trajectory groups

Across the blood pressure trajectory groups, more than 41% of those with CVH SBP and CVH DBP were refugees (*P* = 0.851 and 0.165, respectively, Table 1). Additionally, 88.8% and 92.1% of those with CVH SBP and CVH DBP were unemployed. Besides, more than 86% of both have no stable monthly income. Among those clinically diagnosed with hypertension, 24.4% were non-compliant with anti-hypertensive medication (51.3% and 46.3% in CVH SBP and CVH DBP, respectively). There was no statistical difference in all the sociodemographic factors except gender among SBP trajectory groups (Table 1).

**Table 1:** Characteristics of the study participants of the mid-aged and older Palestinian adults living in Gaza.

**A. Trajectory group of SBP**	**Total**	**Normal-stable**	**High-stable**	**Constant very-high**	***P*-value**
	***N** =* **1,000**	***n** =* **549**	***n** =* **227**	***n** =* **224**	
Age, years (mean ± SD)	59.2 ± 7.5	58.9 ± 7.7	59.7 ± 7.2	59.3 ± 7.6	0.333
Household size, person (mean ± SD)	6.9 ± 1.5	6.9 ± 1.5	6.9 ± 1.5	6.8 ± 1.5	0.623
Gender					0.039^*^
Male	532 (53.2%)	296 (53.9%)	132 (58.2%)	104 (46.4%)	
Female	468 (46.8%)	253 (46.1%)	95 (41.8%)	120 (53.6%)	
Study region					0.897
North	220 (22.0%)	127 (23.1%)	48 (21.1%)	45 (20.1%)	
Gaza	122 (12.2%)	59 (10.8%)	33 (14.5%)	30 (13.4%)	
Midzone	214 (21.4%)	117 (21.3%)	47 (20.7%)	50 (22.3%)	
Khan Younis	223 (22.3%)	123 (22.4%)	48 (21.2%)	52 (23.2%)	
Rafah	221 (22.1%)	123 (22.4%)	51 (22.5%)	47 (21.0%)	
Refugees	422 (42.2%)	236 (43.0%)	93 (41.0%)	93 (41.5%)	0.851
Marital status					0.473
Married	844 (84.4%)	456 (83.1%)	191 (84.1%)	197 (88.0%)	
Non-married	156 (15.6%)	93 (16.9%)	36 (15.9%)	27 (12.0%)	
Education^*^					0.614
Low	451 (45.1%)	257 (46.8%)	99 (43.6%)	95 (42.4%)	
Moderate	295 (29.5%)	154 (28.1%)	74 (32.6%)	67 (29.9%)	
High	254 (25.4%)	138 (25.1%)	54 (23.8%)	62 (27.7%)	
Employment					0.936
Employed	117 (11.7%)	66 (12.0%)	26 (11.4%)	25 (11.2%)	
Non-employed	883 (88.3%)	483 (88%)	201 (88.6%)	199 (88.8%)	
Household income					0.858
<500 NIS	51 (5.1%)	25 (4.5%)	15 (6.6%)	11 (4.9%)	
500–1,000 NIS	41 (4.1%)	25 (4.5%)	9 (4.0%)	7 (3.1%)	
>1,000 NIS	54 (5.4%)	34 (6.2%)	11 (4.8%)	9 (4.0%)	
No stable income	854 (85.4%)	465 (84.8%)	192 (84.6%)	197 (88.0%)	
BMI (Kg/m^2^)	28.2 ± 3.7	28.0 ± 3.7	28.5 ± 3.8	28.1 ± 3.4	0.269
Physical activity					0.382
Low	654 (65.4%)	355 (64.6%)	144 (63.4%)	155 (69.2%)	
Moderate	247 (24.7%)	133 (24.3%)	60 (26.5%)	54 (24.1%)	
High	99 (9.9%)	61 (11.1%)	23 (10.1%)	15 (6.7%)	
Regular cigarette smokers	447 (44.7%)	250 (55.9%)	101 (22.6%)	96 (21.5%)	0.792
Antihypertensive drugs					0.485
ACE inhibitors	23 (2.3%)	-	12 (5.3%)	11 (4.9%)	
Diuretics	109 (10.9%)	-	54 (23.7%)	55 (24.5%)	
Either	75 (7.5%)	-	32 (14.1%)	43 (19.3%)	
Neither	549 (54.9%)	549 (54.9%)	-	-	
Non-compliant	244 (24.4%)	-	129 (56.9%)	115 (51.3%)	
**B. Trajectory group of DBP**	**Total**	**Normal-stable**	**High-stable**	**Moderate to high**	**Elevated to decreasing**	**Constant-very-high**	***P*** **-value**
	***N** =* **1,000**	***n** =* **526**	***n** =* **99**	***n** =* **64**	***n** =* **97**	***n** =* **214**	
Age, years (mean ± SD)	59.2 ± 7.5	58.8 ± 7.7	59.0 ± 6.8	60.1 ± 6.8	59.6 ± 7.2	59.4 ± 7.8	0.686
Household size, person (mean ± SD)	6.9 ± 1.5	6.9 ± 1.5	7.2 ± 1.6	6.9 ± 1.4	6.8 ± 1.5	6.8 ± 1.5	0.336
Gender							0.055
Male	532 (53.2%)	287 (54.6%)	58 (58.6%)	36 (56.3%)	56 (57.7%)	95 (44.4%)	
Female	468 (46.8%)	239 (45.4%)	41 (41.4%)	28 (43.7%)	41 (42.3%)	119 (55.6%)	
Study region							0.567
North	220 (22.0%)	124 (23.6%)	15 (15.1%)	11 (17.2%)	25 (25.8%)	45 (21.0%)	
Gaza	122 (12.2%)	57 (10.8%)	11 (11.1%)	13(20.3%)	9 (9.3%)	32 (15.0%)	
Midzone	214 (21.4%)	114 (21.7%)	23 (23.2%)	13 (20.3%)	16 (16.5%)	48 (22.4%)	
Khan Younis	223 (22.3%)	112 (21.3%)	26 (26.3%)	15 (23.4%)	23 (23.7%)	47 (22.0%)	
Rafah	221 (22.1%)	119 (22.6%)	24 (24.3%)	12 (18.8%)	24 (24.7%)	42 (19.6%)	
Refugees	422 (42.2%)	227 (43.2%)	31 (31.3%)	25 (39.1%)	46 (47.4%)	93 (43.5%)	0.165
Marital status							0.765
Married	844 (84.4%)	437 (83.1%)	82 (82.8%)	53 (82.8%)	81 (83.5%)	191 (89.3%)	
Non-married	156 (15.6%)	89 (16.9%)	17 (17.2%)	11 (17.2%)	16 (16.5%)	23 (10.7%)	
Education^*^							0.658
Low	451 (45.1%)	245 (46.6%)	46 (46.5%)	30 (46.9%)	41 (42.3%)	89 (41.6%)	
Moderate	295 (29.5%)	149 (28.3%)	33 (33.3%)	16 (25.0%)	34 (35.0%)	63 (29.4%)	
High	254 (25.4%)	132 (25.1%)	20 (20.2%)	18 (28.1%)	22 (22.7%)	62 (29.0%)	
Employment							0.361
Employed	117 (11.7%)	64 (12.2%)	14 (14.1%)	8 (12.5%)	14 (14.4%)	17 (7.9%)	
Non-employed	883 (88.3%)	462 (87.8%)	126 (85.9%)	56 (87.5%)	83 (85.6%)	197 (92.1%)	
Household income							0.977
<500 NIS	51 (5.1%)	25 (4.7%)	4 (4.0%)	5 (7.8%)	5 (5.2%)	12 (5.6%)	
500–1,000 NIS	41 (4.1%)	22 (4.2%)	5 (5.1%)	1 (1.6%)	6 (6.2%)	7 (3.3%)	
>1,000 NIS	54 (5.4%)	32 (6.1%)	6 (6.1%)	2 (3.1%)	4 (4.1%)	10 (4.6%)	
No stable income	854 (85.4%)	447 (85.0%)	84 (84.8%)	56 (87.5%)	82 (84.5%)	185 (86.5%)	
BMI (Kg/m^2^)	28.2 ± 3.7	28.0 ± 3.7	28.5 ± 3.9	28.2 ± 3.5	28.3 ± 4.1	28.2 ± 3.3	0.805
Physical activity							0.689
Low	654 (65.4%)	64 (64.6%)	39 (60.9%)	67 (69.1%)	145 (67.8%)	145 (67.8%)	
Moderate	247 (24.7%)	28 (28.3%)	16 (25.0%)	23 (23.7%)	52 (24.3%)	52 (24.3%)	
High	99 (9.9%)	7 (7.1%)	9 (14.1%)	7 (7.2%)	17 (7.9%)	17 (7.9%)	
Regular cigarette smokers	447 (44.7%)	40 (40.4%)	31 (48.4%)	36 (37.1%)	98 (45.8%)	98 (45.8%)	0.430
Antihypertensive drugs							0.106
ACE inhibitors	23 (2.3%)	8 (8.1%)	3 (4.7%)	4 (4.1%)	10 (4.6%)	10 (4.6%)	
Diuretics	109 (10.9%)	18 (18.2%)	12 (18.7%)	29 (30.0%)	58 (27.1%)	58 (27.1%)	
Either	75 (7.5%)	9 (9.1%)	8 (12.5%)	17 (17.5%)	47 (22.0%)	47 (22.0%)	
Neither	549 (54.9%)	-	-	-	-	-	
Non-compliant	244 (24.4%)	64 (64.6%)	41 (64.1%)	47 (48.4%)	99 (46.3%)	99 (46.3%)	

The associations were studied using ANOVA and χ^2^ test.

SBP, Systolic blood pressure; DBP, Diastolic blood pressure; NIS, New Israeli Shekel; BMI, Body Mass Index; ACE inhibitors, Angiotensin-converting enzyme.

^*^Education Level (low: secondary or more subordinate, moderate: diploma or 2 years after high school, and high: bachelor's and above).

Trajectory groups of SBP: (1) Normal-stable (54.9 %): had a stable SBP at 120 mmHg. (2) High-stable (22.7 %): had a stable SBP at 150 mmHg. (3) Constant-very-high (22.4%): SBP was above 160 mmHg.

Trajectory groups of DBP: (1) Normal-stable (52.6 %): had a stable DBP at 80 mmHg. (2) High-stable (9.9%): had a stable DBP at 86 mmHg. (3) Moderate to high (6.4%): had a rapid gain of nearly 10 mmHg from DBP at 85 mmHg and then decreased by almost 5 mmHg but within the high level of DBP. (4) Elevated to decreasing (9.7%): had a rapid decrease of nearly 15 mmHg from DBP at 100 mmHg. (5) Constant-very-high (21.4%): DBP was above 95 mmHg.

* *P*-value < 0.05, statistically significant.

### 3.2. BP trajectories

**Figure 1:**
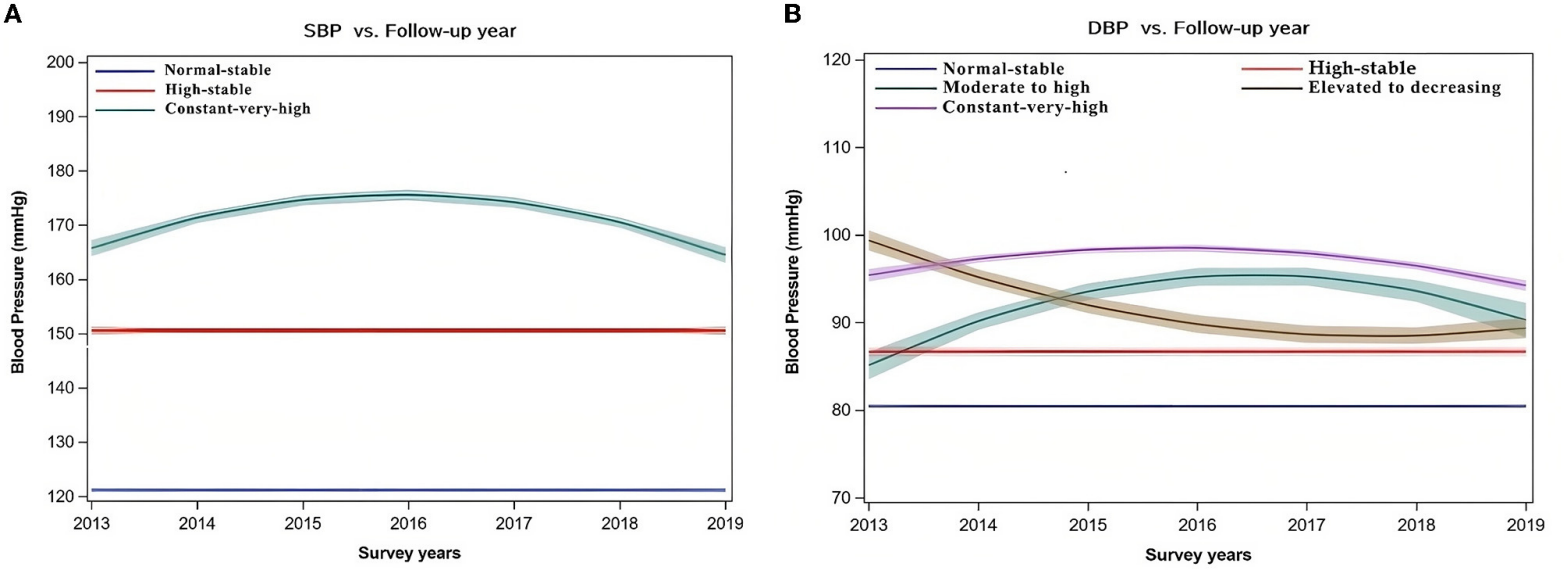
Trajectory modeling of BP of the mid-aged and older Palestinian adults living in Gaza, from 2013 to 2019 (*N* = 1,000). Trajectory groups of SBP: (1) Normal-stable (54.9 %): stable SBP at 120 mmHg. (2) High-stable (22.7 %): had a stable SBP at 150 mmHg. (3) Constant very high (22.4%): had a rapid gain of nearly 10 mmHg from SBP at 165 mmHg and then decreased but within the high level of SBP. Trajectory groups of DBP: (1) Normal-stable (52.6 %): had a stable DBP at 80 mmHg. (2) High-stable (9.9%): had a stable DBP at 86 mmHg. (3) Moderate to high (6.4%): had a rapid gain of nearly 10 mmHg from DBP at 85 mmHg and then decreased by almost 5 mmHg but within the constant high level of DBP. (4) Elevated to decreasing (9.7%): had a rapid decrease of nearly 15 mmHg from DBP at 100 mmHg. (5) Constant very high (21.4%): had a gain of nearly 3 mmHg from DBP at 95 mmHg and decreased but within the constant high level of DBP.

Three distinct trajectory changes of SBP and five DBP were identified, with 13,978 measurements of SBP and DBP (from 2013 to 2019) for our 1,000 participants (Figure 1). The three SBP trajectory groups were: Group 1 (Normal-stable, 54.9%), Group 2 (High-stable, 22.7%), and Group 3 (Constant-very-high, CVH, 22.4%). In most years, SBP was above 160 mmHg in the CVH group.

Among the five DBP trajectory groups (normal-stable, high-stable, moderate to high, elevated to decreasing, and CVH), the prevalence of CVH DBP was 21.4%. From 2013 to 2019, DBP was above 95 mmHg in the CVH DBP group.

Figure 1 displays the definition of each BP trajectory group based on their follow-up data: The “Normal-stable” had a stable SBP at 120 mmHg or stable DBP at 80 mmHg, “High-stable” had a stable SBP at 150 mmHg or a stable DBP at 86 mmHg, “Moderate to high” had a rapid gain of nearly 10 mmHg from DBP at 85 mmHg and then decreased by almost 5 mmHg but within a high level of DBP, and “Elevated to decreasing” had a rapid decrease of nearly 15 mmHg from DBP at 100 mmHg.

### 3.3. Characteristics across BP trajectory groups

Across the BP trajectory groups, more than 86% of CVH SBP and CVH DBP have no stable monthly income among those clinically diagnosed with hypertension, 24.4% were non-compliant with antihypertensive medication (51.3 and 46.3% in CVH SBP, and CVH DBP, respectively). Individuals with hypertension were more likely to use diuretic drugs than angiotensin-converting enzyme (ACE) inhibitors (10.9 vs. 2.3%), whereas 7.5 % take ACE inhibitors or diuretic drugs. There was no difference in all the sociodemographic factors except gender among the different BP trajectory groups.

### 3.4. Distribution of war-related traumatic events by trajectories of DBP and DBP

The overall prevalence of self-reported injury (or family member), death of a family member, violence due to house bombing, and living in debt was 51.4, 54.1, 66.5, and 65.1%, respectively (Table 2). The prevalence of self-reported injury or their family member and death of a family member during wars was 63.8 and 65.6% for CVH SBP (*N* = 224). The corresponding figures were 66.4 and 66.4% for CVH DBP (*N* = 214).

**Table 2:** Distribution of experiencing war-related traumatic events on the trajectory of systolic and diastolic BP of the mid-aged and older Palestinian adults living in Gaza, from 2013 to 2019 (*N* = 1,000).

**War-related traumatic events^¶^**	**Total**	**Trajectory group of SBP**			***P*-value**	**Trajectory group of DBP**					***P*-value**
		**Normal-stable**	**High-stable**	**Constant-very-high**		**Normal-stable**	**High-stable**	**Moderate to high**	**Elevated to decreasing**	**Constant-very-high**	
	***N** =* **1,000**	***n** =* **549**	***n** =* **227**	***n** =* **224**		***n** =* **526**	***n** =* **99**	***n** =* **64**	***n** =* **97**	***n** =* **214**	
1) Injury of any family member	514 (51.4%)	278 (50.6%)	93 (41.0%)	143 (63.8%)	<0.001^*^	269 (51.1%)	48 (48.5%)	18 (28.1%)	37 (38.1%)	142 (66.4%)	<0.001^*^
2) Death of any family member	541 (54.1%)	279 (50.8%)	115 (50.6%)	147 (65.6%)	<0.001^*^	269 (51.1%)	48 (48.5%)	32 (50.0%)	50 (51.5%)	142 (66.4%)	0.002^*^
3) Serious illness of any family member	303 (30.3%)	154 (28.1%)	76 (33.5%)	73 (32.6%)	0.228	146 (27.7%)	33 (33.3%)	23 (35.9%)	34 (35.1%)	67 (31.3%)	0.387
4) Loss of job	137 (13.7%)	79 (14.4%)	31 (13.7%)	27 (12.1%)	0.693	74 (14.1%)	14 (14.1%)	12 (18.8%)	14 (14.4%)	23 (10.8%)	0.545
5) Business Bankruptcy	73 (7.3%)	35 (6.4%)	19 (8.4%)	19 (8.5%)	0.463	32 (6.1%)	6 (6.1%)	10 (15.6%)	10 (10.3%)	15 (7.0%)	0.055
6) Exposure to violence due to house bombing	665 (66.5%)	362 (65.9%)	138 (60.8%)	165 (73.6%)	0.014^*^	347 (66.0%)	70 (70.7%)	40 (62.5%)	46 (47.4%)	162 (75.7%)	<0.001^*^
7) Living in debt	651 (65.1%)	326 (59.4%)	151 (66.5%)	174 (77.7%)	<0.001^*^	312 (59.3%)	69 (69.7%)	37 (57.8%)	68 (70.1%)	165 (77.1%)	<0.001^*^
8) Marital separation or divorce	59 (5.9%)	26 (4.7%)	21 (9.3%)	12 (5.4%)	0.049^*^	27 (5.1%)	6 (6.1%)	4 (6.3%)	9 (9.3%)	13 (6.1%)	0.630
9) Major disasters of any family member	149 (14.9%)	79 (14.4%)	38 (16.7%)	32 (14.3%)	0.675	74 (14.1%)	15 (15.2%)	13 (20.3%)	18 (18.6%)	29 (13.5%)	0.541
Composite score^*^ (Mean ± SD)	−0.16 ± 0.09	−0.10 ± 0.07	−0.19 ± 0.11	0.44 ± 0.10	<0.001^*^	−0.10 ± 0.08	−0.00 ± 0.17	−0.39 ± 0.19	−0.31 ± 0.15	0.49 ± 0.11	<0.001^*^

^¶^Variables were recoded binary (yes or no).

^*^Principal component analysis (PCA) was used to develop the composite score based on nine war-related traumatic events.

^*^P-value < 0.05, statistically significant.

Moreover, the composite score based on the overall events indicated that CVH for SBP and DBP trajectory changes have the highest adverse event than other trajectory groups. The mean score for CVH SBP and DBP trajectories was 0.44 ± 0.10 and 0.49 ± 0.11, respectively (*P* < 0.001) (Table 2). See Supplementary Table 1 of factor loadings of PCA based on the war-related traumatic events.

### 3.5. Association between traumatic war events and BP trajectories

Multinomial logistic regression analysis revealed that war-related traumatic events were significantly associated with the trajectory groups of CVH SBP and DBP (Table 3). After adjusting for covariates, self-reported injury or their family member, death of a family member during wars were positively associated with CVH SBP with an odds ratio [95 CI, OR = 1.79 (1.28–2.48) and 1.90 (1.36–2.65)], respectively. The corresponding figures were [95 CI, OR = 1.92 (1.36–2.71) and 1.90 (1.35–2.68)] for CVH DBP (Table 3).

**Table 3:** Association between war-related traumatic events and trajectory of systolic and diastolic BP of the mid-aged and older Palestinian adults living in Gaza, from 2013 to 2019 (*N* = 1, 000).

**War-related traumatic events^¶^**	**Trajectory group of SBP**			
	**High-stable**		**Constant-very-high**	
	**Crude OR (95% CI)**	**Adjusted OR (95% CI)**	**Crude OR (95% CI)**	**Adjusted OR (95% CI)**
1) Injury of any family member	0.68 (0.49–1.03)	0.73 (0.51–1.08)	1.72 (1.25–2.37)^*^	1.79 (1.28–2.48)^*^
2) Death of any family member	0.99 (0.73–1.35)	1.03 (0.75–1.42)	1.85 (1.34–2.55)^*^	1.90 (1.36–2.65)^*^
3) Serious illness of any family member	1.29 (0.93–1.80)	1.31 (0.93–1.85)	1.24 (0.89–1.73)	1.23 (0.87–1.74)
4) Loss of job	0.94 (0.60–1.47)	0.96 (0.61–1.52)	0.82 (0.51–1.30)	0.86 (0.53–1.35)
5) Business Bankruptcy	1.34 (0.75–2.40)	1.35 (0.75–2.45)	1.36 (0.76–2.43)	1.36 (0.75–2.49)
6) Exposure to violence due to house bombing	0.80 (0.58–1.10)	0.81 (0.58–1.12)	1.44 (1.02–2.04)^*^	1.44 (1.01–2.05)^*^
7) Living in debt	1.36 (0.98–1.88)	1.37 (0.98–1.91)	2.38 (1.66–3.40)^*^	2.49 (1.73–3.60)^*^
8) Marital separation or divorce	0.92 (0.66–1.27)	1.21 (0.86–1.64)	0.88 (0.44–1.77)	0.83 (0.40–1.73)
9) Major disasters of any family member	1.19 (0.77–1.83)	1.20 (0.78–1.85)	0.99 (0.64–1.54)	1.01 (0.66–1.59)
**War-related traumatic events** ^¶^	**Trajectory group of DBP**							
	**High-stable**		**Moderate to high**		**Elevated to decreasing**		**Constant-very-high**	
	**Crude OR (95% CI)**	**Adjusted OR (95% CI)**	**Crude OR (95% CI)**	**Adjusted OR (95% CI)**	**Crude OR (95% CI)**	**Adjusted OR (95% CI)**	**Crude OR (95% CI)**	**Adjusted OR (95% CI)**
1) Injury of any family member	0.89 (0.59–1.38)	0.96 (0.62–1.50)	0.78 (0.59–1.09)	0.81 (0.63–1.16)	0.95 (0.61–1.49)	0.99 (0.62–1.55)	1.88 (1.35–2.62)^*^	1.92 (1.36–2.71)^*^
2) Death of any family member	0.90 (0.59–1.38)	0.95 (0.61–1.49)	0.95 (0.57–1.61)	1.02 (0.60–1.76)	1.02 (0.66–1.56)	1.02 (0.65–1.61)	1.80 (1.33–2.58)^*^	1.90 (1.35–2.68)^*^
3) Serious illness of any family member	1.30 (0.82–2.06)	1.33 (0.83–2.14)	1.46 (0.85–2.52)	1.49 (0.85–2.61)	1.40 (0.89–2.22)	1.40 (0.88–2.24)	1.19 (0.84–1.68)	1.20 (0.84–1.71)
4) Loss of job	1.01 (0.54–1.86)	1.01 (0.54–1.88)	1.41 (0.72–2.77)	1.44 (0.71–2.88)	1.03 (0.55–1.91)	1.04 (0.57–1.94)	0.74 (0.45–1.21)	0.75 (0.44–1.23)
5) Business Bankruptcy	1.00 (0.41–2.45)	1.02 (0.41–2.49)	2.86 (1.33–4.16)^*^	2.87 (1.26–4.20)^*^	1.77 (0.84–3.74)	1.85 (0.86–3.96)	1.16 (0.61–2.21)	1.17 (0.62–2.24)
6) Exposure to violence due to house bombing	1.24 (0.78–1.99)	1.29 (0.80–2.08)	0.85 (0.50–1.47)	0.85 (0.49–1.49)	0.94 (0.60–1.47)	0.96 (0.60–1.49)	1.59 (1.11–2.30)^*^	1.62 (1.13–2.38)^*^
7) Living in debt	1.58 (0.99–2.51)	1.65 (1.03–2.66)^*^	0.94 (0.55–1.59)	0.95 (0.56–1.61)	1.61 (1.01–2.57)^*^	1.69 (1.05–2.73)^*^	2.35 (1.63–3.41)^*^	2.37 (1.63–3.45)^*^
8) Marital separation or divorce	0.84 (0.34–2.09)	0.94 (0.34–2.57)	0.81 (0.27–2.39)	0.82 (0.27–2.52)	0.53 (0.24–1.16)	0.48 (0.22–1.13)	0.84 (0.42–1.65)	0.82 (0.40–1.66)
9) Major disasters of any family member	1.09 (0.59–1.99)	1.13 (0.61–2.09)	1.55 (0.81–3.00)	1.58 (0.80–3.14)	1.39 (0.78–2.45)	1.45 (0.80–2.63)	0.96 (0.60–1.52)	0.94 (0.57–1.50)

Multinomial logistic regression analysis was conducted. The reference group was normal-stable SBP. **The models** adjusted for age, gender, region, family size, citizenship, marital status, income, education, employment, smoking, physical activity, body mass index, fruits and vegetable intake, type 2 diabetes, and lipid profile (total cholesterol, triglycerides, HDL-C, and LDL-C).

^¶^Variables were recoded binary (yes or no).

^*^P-value <0.05, statistically significant association.

Moreover, living in debt was positively associated with CVH SBP with an odds ratio [95 CI, OR = 2.49 (1.73–3.60) and CVH DBP with an odds ratio [95 CI, OR = 2.37 (1.63–3.45)] (Table 3), suggesting that they were 2.49 and 2.37 times more likely to have CVH SBP and CVH DBP.

## 4. Discussion

This study investigated the trajectory of BP using longitudinal data collected from a uniquely vulnerable population, the Palestinian adults living in Gaza Strip; 64% of them are refugees. They have faced adverse and challenging living conditions, including war trauma. Our findings show that exposure to frequent traumatic events is linked to chronic disease risks, as indicated by elevated BP over time. In our trajectory analyses of SBP and DBP measurements (2013–2019), 22.4 and 21.4% of participants had CVH SBP (>160 mmHg) and DBP (>95 mmHg). The war-related traumatic events were positively associated with adverse BP trajectories.

This study provides evidence of the ongoing hypertension burden and the particular difficulties this vulnerable population faces. The exposure to war-related traumatic events might cause a double or triple burden on many families in Gaza. We may find Palestinians in Gaza from the same family who have been injured, killed, or even exposed to violence due to the bombing of their houses and others. The burden of the presence of all these events caused by wars might increase the frequency of developing hypertension or lead to worsening systolic and diastolic BP levels more and more. The Palestinians in Gaza are greatly affected by events surrounding oscillating stability and cumulative traumatic life (8). It is hypothesized that living in a situation of ongoing trauma may lead to further suffering in an agitated environment, leaving them vulnerable to developing hypertension. The highest trajectory change “CVH” of SBP and DBP, characterized by a rapid gain of BP values, throughout the follow-up (2013–2019), within a constant high level of SBP and DBP, these are serious indicators. The presence of high affliction of war-related traumatic events among those people indicates a significant association of war-related traumatic events, which are: injury of any family member, exposure to violence due to house bombing, and living in debt, with the highest trajectory change “CVH” of SBP and DBP. The relatively high proportion of “CVH” trajectory change (22.4%) in SBP and (21.4%) in DBP confirm that large numbers of hypertension patients could be affected by war-related traumatic events, which is a significant concern and should be taken into consideration in the prevention/management of NCDs.

We found injury due to wars was positively associated with adverse BP trajectories. The finding is in line with another study, which reported that initial injury severity was independently associated with hypertension (33). Another study conducted in Iraq and Afghanistan veterans reported that the severity of combat injury was associated with the subsequent development of hypertension, coronary artery disease, diabetes mellitus, and chronic kidney disease (34).

Moreover, Gaza Strip is a complicated environment with structural challenges such as the lack of medicines, low salaries, and specialized training abroad (35). The healthcare providers in Gaza are barred from accessing training and professional development opportunities outside Gaza due to wars and conflicts there (36). It challenges the health system's capacity to respond to the population's basic needs (37). Due to those wars' repercussions, there is a noticeable shortage of medicines for cancer and chronic diseases. According to the Ministry of Health in Gaza, over 50% of the essential medications for chronic diseases were unavailable (38). In light of those mentioned earlier, the presence of 24.4% of the diagnosed hypertension being non-compliant with antihypertensive medication in our study is reasonable; these findings support the figures of a survey that was undertaken at the outpatient clinics of the Ministry of Health in West Bank, Palestine (*n* = 450), which indicated that more than half of the hypertension patients (54.2%) had poor adherence with medications, where one of the reasons for the low-adherence to medications was cost and unavailability of these medications at the healthcare centers (39).

Various potential risk factors, including psychosocial factors and stressful experiences in war life in Gaza, may play a role in developing hypertension (40). Frequent exposure to stressful events is thought to be one of the most common environmental causes of hypertension on a physiological basis (41). Studies have shown that those stressful events induce various neurochemical, neurotransmitter, and hormonal changes, predominantly by triggering the sympathetic nervous system (SNS) and hypothalamic-pituitary-adrenal (HPA) axes (42). SNS and HPA axes are woken up to release chemical mediators to protect the body from stress (43). This is in line with a cohort study of 122.816 adults aged ≥30 years in a different cultural and socioeconomic setting in France, where perceived stress was significantly associated with high BP (44).

Future research will help explain the biological mechanisms for the effect of war events on BP. A study suggests that our body creates a surge of hormones in a stressful environment, which then causes the heart to beat faster and blood vessels to narrow (45). Stability and living in peace without wars can help people with hypertension trigger the relaxation response and reduce stress. Thus, reducing the risk of CVDs.

The major strength of this study is targeting a uniquely vulnerable population, the mid-aged and older adults, who face adverse and challenging living conditions and war trauma. It is the first study to investigate the longitudinal trajectory of SBP and DBP in Gaza, a special place in the world. This study involved cross-sectional and historical longitudinal designs with repeated SBP and DBP measurements for a representative population, enabling us to perform the trajectory analyses.

This study has limitations. First, our data collection was after the wars. Although a causal relationship cannot be made due to the factor that war-related traumatic events were collected in 2019, reverse causation is unlikely. Second, we could not know the likelihood of developing BP among exposed groups by not including a control group. Third, we cannot rule out selection bias, as only those who regularly visited the primary healthcare centers in Gaza Strip were enrolled in the study. Fourth, we did not include all age groups of adults. Fifth, the sample size and study duration are limited.

Future efforts are needed to enhance the implementation of care models to improve NCD management, including hypertension, health education, and medication services in Gaza Strip. In addition, an in-depth trauma care system evaluation is needed in Gaza's health system. Therefore, a comprehensive intervention toward war-related traumatic events should be provided to the people in Gaza Strip.

In conclusion, this study provides evidence that the burden of war-related traumatic events among mid-aged and older Palestinian adults living in Gaza Strip is high. Such war-related traumatic events are positively associated with adverse BP trajectories. Efforts and sustainable programs are needed to enhance healthcare services to improve health education and medication services and for NCD prevention and management, including hypertension, mental health, and other prevalent health conditions in Gaza Strip.

## Data availability statement

The raw data supporting the conclusions of this article will be made available by the authors, without undue reservation.

## Ethics statement

The studies involving human participants were reviewed and approved by the Helsinki Ethical Approval Committee affiliated with the Palestinian Health Research Council (PHRC) in Gaza and the Research Department at the Department of Human Resources Development, Ministry of Health, Gaza (PHRC/HC/576/19). The patients/participants provided their written informed consent to participate in this study. Written informed consent was obtained from the individual(s) for the publication of any potentially identifiable images or data included in this article.

## Author contributions

MJ, XL, and YW designed the study. MJ collected the data, made data entries with Gaza's teamwork, analyzed the data, and drafted the manuscript. MM assisted data analysis, interpretation of results, and manuscript drafting. XL, ZS, and YW designed the research hypothesis and guided data analysis. MB analyzed the trajectory data and assisted in the data interpretation and editing. HA helped in data collection and entry. YW provided administration support for the study and is the guarantor of this work. All authors revised the manuscript and approved the final version to be submitted. This work is part of MJ's PhD dissertation research in Xi'an Jiaotong University.
