# Supplementary material for: Association between war-related traumatic events and blood pressure trajectory: a population-based study among the mid-aged and older Palestinian adults living in Gaza
Source: Front Public Health. 2023 Jun 15;11:1073284. doi: 10.3389/fpubh.2023.1073284 (PMC10310537; doi:10.3389/fpubh.2023.1073284)
Supplement: Supplementary file 1 [file Table_1.DOCX]

**Supplemental Table 1. Factor loadings of principal component analysis (PCA) based on the war-related traumatic events**

| Factor loadings | | | War-related traumatic events |
| --- | --- | --- | --- |
| Component 3 | **Component 2** | **Component 1** |  |
| -0.0235 | 0.0011 | 0.5291 | **1. Injury of any family member** |
| -0.0331 | 0.0150 | 0.5239 | **2. Death of any family member** |
| -0.1940 | 0.6094 | 0.0343 | **3. Serious illness of any family member** |
| -0.3732 | 0.4647 | 0.0124 | **4. Loss of job** |
| 0.4980 | 0.3862 | 0.0072 | **5. Business Bankruptcy** |
| 0.0551 | -0.0369 | 0.5018 | **6. Exposure to violence due to house bombing** |
| 0.0585 | -0.0636 | 0.4375 | **7. Living in debt** |
| 0.7053 | -0.0394 | -0.0278 | **8. Marital separation or divorce** |
| 0.2635 | 0.5063 | 0.0145 | **9. Major disasters of any family member** |
