# Supplementary material for: Association between war-related traumatic events and blood pressure trajectory: a population-based study among the mid-aged and older Palestinian adults living in Gaza
Source: Front Public Health. 2023 Jun 15;11:1073284. doi: 10.3389/fpubh.2023.1073284 (PMC10310537; doi:10.3389/fpubh.2023.1073284)
Supplement: Supplementary file 2 [file Table_2.DOCX]

**Part I)** A cross-sectional study (2019)

The repeated anthropometric and biochemical measurements i.e., SBP & DBP.

*Main questionnaire parts*

*Data extracted*

**Part II)** Historical longitudinal data (2013-2019)

Extracting from the existing data

Sociodemographic, medical history, behavioral lifestyle, and unique issues of war-related traumatic events.

Face to face interview

**Supplemental Figure 1. Fellow chart of the study design**
